# Primary Breast Angiosarcoma: A Report of a Rare Case

**DOI:** 10.7759/cureus.109782

**Published:** 2026-05-27

**Authors:** Chalent Alexakis, Annezo Marinatou, Evgenia Kalogridaki, Eftychia Katagi, Panagiotis Daskalakis

**Affiliations:** 1 Rural Medicine, General Hospital of Thebes, Thebes, GRC; 2 Surgery, Breast Unit, General Maternal Hospital of Athens ‘Elena Venizelou’, Athens, GRC; 3 Obstetrics and Gynecology, General Maternal Hospital of Athens ‘Elena Venizelou’, Athens, GRC

**Keywords:** angiosarcoma, breast cancer, breast surgery, local excision, tumor imaging

## Abstract

Breast angiosarcomas are a rare type of cancer, comprising only a small percentage of all malignant breast tumors. They can be divided into two main types: primary angiosarcoma, which develops in the absence of any pre-existing condition, and secondary angiosarcoma, which most often arises in previously irradiated breast tissue or following any other disorders affecting the breast. The primary one is an aggressive tumor, whereas the secondary may behave differently due to its relation with previous treatments. These differences need to be distinguished for the correct diagnosis and effective management. A 71-year-old woman with a personal history of hypothyroidism and benign breast lumpectomies presented to our clinic with a right breast mass. Initial imaging suggested a fibroadenoma, but after the excision of the tumor, it was identified as grade III angiosarcoma. The patient declined mastectomy, choosing local excision and surveillance.

## Introduction

Angiosarcomas are very rare, accounting for less than 1% of all breast malignancies. They are divided into two categories: primary angiosarcoma, which represents a very rare histological type of breast cancer, and secondary breast angiosarcoma [1]. Primary breast angiosarcoma is a malignant vascular neoplasm of the breast, originating from the endothelial cells of the vessels within the breast parenchyma. The first recorded reference to breast angiosarcoma is believed to date back to 1887, when Schmidt studied and documented 11 cases of this disease [2].

Primary angiosarcoma of the breast (PBA) mainly concerns the nipple and appears as a tumor of the gland without skin lesions [3]. Very rarely, cases have been described involving male patients [4]. More often, the tumor has dimensions ranging from 5.5 cm to 7 cm, while lesions smaller than 2 cm are very rare [5]. It mainly affects young patients aged 30-50 [5]. In the case that concerns both breasts, bilaterally, then the second location is considered local regional metastasis.

In PBA, no grading system of clinical significance has been established. A three-level classification system was used in the past; however, it was abandoned as no correlation of grade of malignancy with patient prognosis was observed.

Due to the rarity of the disease and the nonspecific clinical and imaging findings, the diagnosis remains demanding and may lead to misdiagnosis. The aim of this case report is to present a rare case of PBA in a 71-year-old woman and to highlight the diagnostic and therapeutic challenges associated with this uncommon malignancy. 

## Case presentation

A 71-year-old female patient presented to our clinic after a new ultrasound finding during her annual breast screening. Her past medical history includes hypothyroidism, managed with levothyroxine 75 mcg, and osteoporosis. Her past surgical history includes a hysterectomy and excision of four breast lesions, one in the left and three on the right breast, all of which were diagnosed as sclerosing adenosis. She is a non-smoker, does not consume alcohol, and is independent in her daily activities. Her family history is notable for a cousin diagnosed with esophageal cancer at the age of 62 years.

On examination, a palpable mass was detected in the upper outer quadrant of the right breast. The examination of the contralateral breast was unremarkable, with no lymphadenopathy bilaterally. Breast mammogram revealed a lipoma in the right breast located at the 12 o’clock position, measuring 1.4 cm, and was classified as BIRADS 2 (Figure 1). On ultrasound, the mass appeared hyperechogenic, located at 12 o'clock, and measured 1.4 cm.

**Figure 1:**
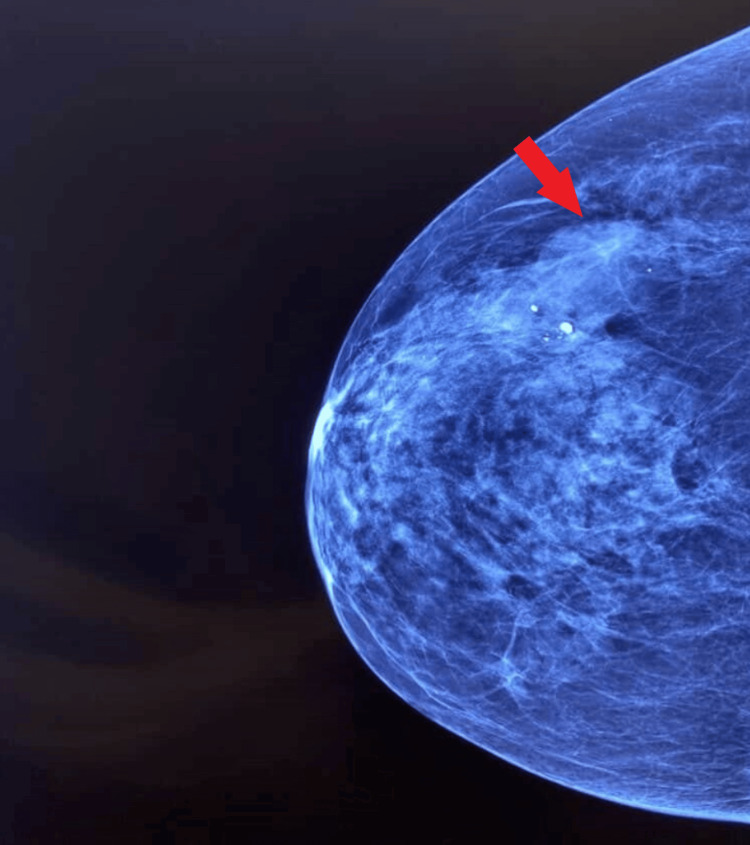
Mammographic image of the right breast in the craniocaudal (CC) view demonstrating a heterogeneously dense breast parenchyma. The red arrow points at the referred lipoma.

The patient was advised to undergo bilateral magnetic resonance mammography (MRM) for further evaluation. The MRM identified a multilobulated formation located in the right anterior upper quadrant, classified as MRM BIRADS 4A, consistent with a fibroadenomatous mass (Figures 2, 3). Although a biopsy would have been the standard approach for a BIRADS 4A lesion, the patient requested complete excision of the tumor, which was performed under local anesthesia via lumpectomy.

**Figure 2:**
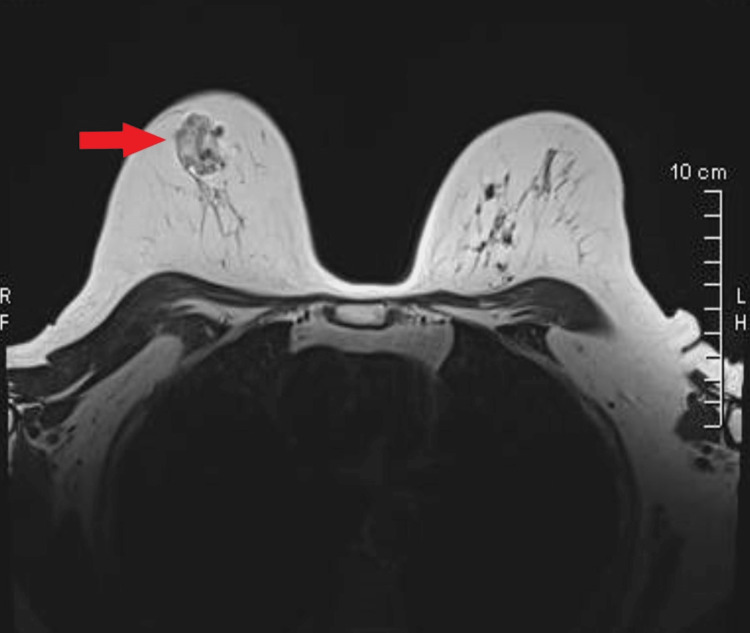
Axial Τ1-weighted breast MRI without fat saturation. Asymmetrical mass in the right breast, hyperintense with irregular margins and surrounding enhancement. Internal signal voids, suggesting vascular structures within the lesion. The red arrow points at the tumor.

**Figure 3:**
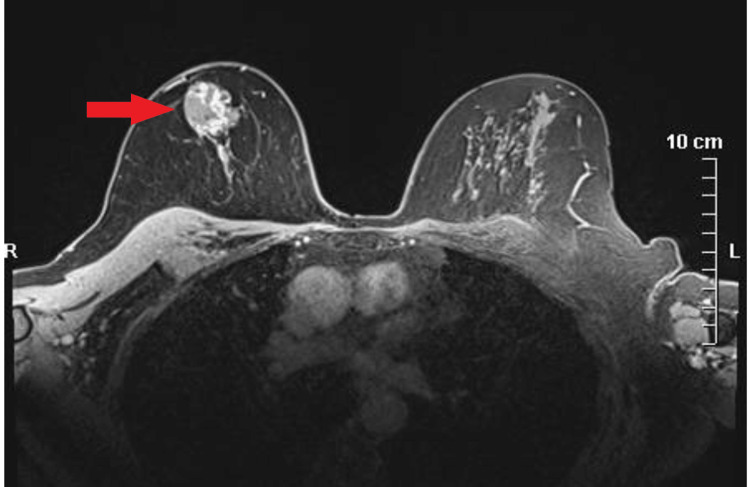
Axial post-contrast T1-weighted fat-suppressed breast MRI. Hyperintense, irregular mass in the right breast with internal vascular structures. The lesion exhibits heterogeneous enhancement. The red arrow points at the lesion.

The histopathological report revealed part of the fibrofatty layer of the breast gland, about 4 cm x 4 cm x 2 cm in size, with the development of a malignant mesenchymal neoplasm exhibiting morphological and immunohistochemical characteristics compatible with grade III angiosarcoma. The surgical margins were reported as clear. The tumor is characterized by anastomosing small and medium-sized vascular spaces within the breast parenchyma, with malignant endothelial cells showing nuclear atypia, mild mitotic activity, and a cell proliferation index of approximately 45%. Immunohistochemical analysis showed the following results: PaCK(-), P53(+), VIM(+), CD34(+), and CD31(+) (Figure 4).

**Figure 4:**
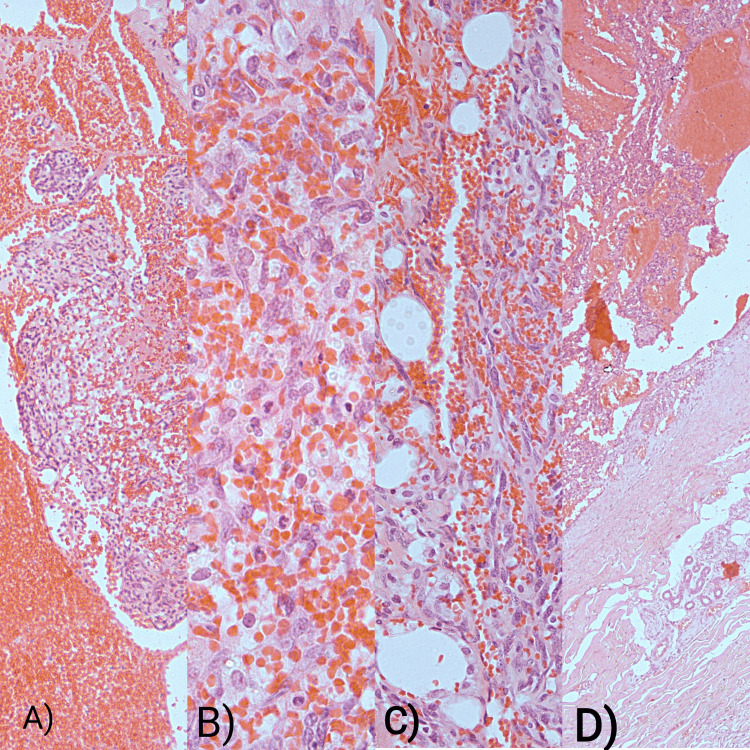
Histologic features of angiosarcoma of the breast (H&E staining) A) Intermediate magnification (H&E, ×10) showing irregular, anastomosing vascular channels and solid cellular areas infiltrating the surrounding stroma B) High-power view (H&E, ×40) showing marked cytologic atypia of malignant endothelial cells with enlarged pleomorphic nuclei, prominent nucleoli and a hemorrhagic background. C) Higher magnification (H&E, ×20) showing atypical endothelial cells with hyperchromatic pleomorphic nuclei lining vascular spaces containing erythrocytes. D) Low-power view (H&E, ×4) showing a haemorrhagic infiltrative vasoformative neoplasm involving the dermis and subcutis.

The tumor board decided on the staging of the patient and re-evaluation. Computed tomography (CT) revealed no distant metastasis, and the PET scan showed no findings compatible with secondary involvement. The complete blood count and biochemical profile were normal. Tumor markers are shown in Table 1.

**Table 1:** Tumor markers CEA: Carcinoembryonic antigen; CA 125: cancer antigen 125

Tumor Marker	Patient Value	Normal Range
CEA	1.5 ng/mL	< 3.0 ng/mL
CA 15-3	32.4 U/mL	< 30 U/mL
CA 125	46.1 U/mL	< 35 U/mL

After histopathological confirmation of the specimen and staging of the patient, the oncology board decided that the best course of treatment would be mastectomy. After informing the patient regarding the treatment plan, she refused and suggested that if the tumor reappeared, it would be locally excised again. The multidisciplinary team determined that the patient should be closely monitored with her annual breast screening, complete blood count, biochemical profile, and chest and abdominal CT for the detection of distant metastasis every six months. After being disease-free for three years, the patient returned with a recurrence, which was locally excised; her screening for metastasis was clear.

## Discussion

Regarding its frequency, primary angiosarcoma is estimated to account for one-quarter of all primary sarcomas of the breast [6]. Its etiology is still unclear, although some correlation has been found with environmental factors due to increased incidence in certain geographic areas [7]. In the study of the pathogenesis of the disease, some research has shown mutations in PLCG1 and KDR, which are involved in the VEGFR2 signaling pathway [8]. For PBA, no grading system with clinical significance has been established. In the past, a three-tier grading system was used, but it was abandoned as no correlation was observed between malignancy grade and patient prognosis.

The imaging studies for the evaluation of a breast mass include mammography, ultrasound, and MRI. Incidental or annual screening imaging is usually responsible for the identification of non-palpable lesions. PBA does not have any typical imaging findings and often mimics other malignancies like invasive carcinoma [9]. On mammography, an irregular noncalcified mass or architectural distortion may be seen, while ultrasound shows an irregular hypoechoic lesion. MRI outlines lesions with an irregular border and heterogeneous internal enhancement. Given their vascular nature, angiosarcomas typically demonstrate low T1 signal intensity and high T2 signal intensity [9].

The morphological features of primary angiosarcomas vary and are characterized either by vascular or solid components [3]. The vessels of the tumor may be dilated or compressed. Endothelial cells contain atypical, hyperchromatic, spindle-shaped nuclei. Well-differentiated angiosarcomas present well-formed vessels with mild endothelial atypia. Irregular vessels with infiltration of malignancy through adipose and breast tissue are present [6]. In contrast, poorly differentiated angiosarcomas are composed of solid sheets of spindle and epithelioid cells intermingled with vascular networks, hematomas, and hemorrhagic necrosis. Hemorrhagic extension outside the tumor boundary indicates extension of malignancy. Angiosarcomas typically express positivity for CD31 and CD34, with possible positivity for ERG, FLI-1, and D2-40 [8]. However, they are negative for progesterone and estrogen receptors, indicating no hormonal influence in their pathogenesis [10].

The primary curative treatment strategy for primary breast angiocarcinoma is complete surgical resection of the tumor with negative margins [6]. Due to the nature of these tumors, which can be very aggressive, mastectomy is considered the preferred surgical procedure [7]. However, until today, there hasn’t been a clinical trial that compares the efficacy of mastectomy versus breast-conserving surgery [10]. In practice, the parameter that influences the course of treatment is the size of the tumor in relation to the size of the breast. Small tumors, where negative margins can be achieved, are usually excised locally while ensuring a good cosmetic outcome [11]. An interesting finding in the literature is that patients treated with local excision had a better overall survival compared to those who underwent mastectomies. However, mastectomies are often reserved for larger and more extensive tumors [11].

Breast angiosarcomas are considered to be a rare entity. Bordoni et al. in 2016 reported that in patients with breast angiosarcoma, regional metastasis to the axillary lymph nodes was found to be uncommon [5]. Furthermore, Herb et al. concluded that the incidence of metastasis to regional lymph nodes was less than 6%.

After surgical excision, chemotherapy plays an important role in the treatment plan. First-line agents used for angiosarcomas include anthracyclines and taxanes [13]. The literature shows that the combination of doxorubicin and ifosfamide results in better overall survival and longer disease-free periods compared to anthracycline alone. This combination seems to be especially useful in patients with locally advanced metastatic angiosarcoma [5].

## Conclusions

We herein present a case of PBA in a 71-year-old woman, who initially presented with nonspecific clinical and imaging findings that led to a diagnostic challenge. PBA is a rare type of breast cancer associated with high rates of local recurrence and distant metastasis. The imaging and clinical features are nonspecific, often leading to misdiagnosis. Surgery, either local excision or mastectomy, is the treatment of choice, depending on the size of the tumor. The role of chemotherapy and radiotherapy in the treatment plan remains an area that requires further study. The aim of presenting this case is to underline the diagnostic challenges and clinical management of this rare entity. In the meantime, we believe that reporting such cases helps raise awareness among breast surgeons to avoid misdiagnoses.

## References

[1] Tan PH, Ellis I, Allison K (2020). The 2019 World Health Organization classification of tumours of the breast. Histopathology.

[2] Tamura G, Kaizuka H, Iwasaki T (1887). About the angiosarcoma of the breast (Article in German). Arch Kim Chir.

[3] Fraga-Guedes C, Gobbi H, Mastropasqua MG, Botteri E, Luini A, Viale G (2012). Primary and secondary angiosarcomas of the breast: a single institution experience. Breast Cancer Res Treat.

[4] Wang ZS, Zhan N, Xiong CL, Li H (2007). Primary epithelioid angiosarcoma of the male breast: report of a case. Surg Today.

[5] Bordoni D, Bolletta E, Falco G (2016). Primary angiosarcoma of the breast. Int J Surg Case Rep.

[6] Adem C, Reynolds C, Ingle JN, Nascimento AG (2004). Primary breast sarcoma: clinicopathologic series from the Mayo Clinic and review of the literature. Br J Cancer.

[7] Cantile M, Di Bonito M, Cerrone M, Pizzolorusso A, Apice G, Botti G, De Chiara A (2018). Primary breast angiosarcoma in young women from the same geographic region in a short period of time: only a coincidence or an increased risk?. Breast J.

[8] Huang SC, Zhang L, Sung YS (2016). Recurrent CIC gene abnormalities in angiosarcomas: a molecular study of 120 cases with concurrent investigation of PLCG1, KDR, MYC, and FLT4 gene alterations. Am J Surg Pathol.

[9] Wienbeck S, Meyer HJ, Herzog A (2017). Imaging findings of primary breast sarcoma: results of a first multicenter study. Eur J Radiol.

[10] Hoda SA, Brogi E, Koerner FC, Rosen PP (2021). Rosen’s Breast Pathology, 5e. https://pathology.lwwhealthlibrary.com/book.aspx?bookid=3003.

[11] Yin M, Wang W, Drabick JJ, Harold HA (2017). Prognosis and treatment of non-metastatic primary and secondary breast angiosarcoma: a comparative study. BMC Cancer.

[12] Herb J, Maduekwe UN, Goel N, Rosenberger LH, Spanheimer PM (2022). Does angiosarcoma of the breast need nodal staging?. J Am Coll Surg.

[13] Young RJ, Natukunda A, Litière S, Woll PJ, Wardelmann E, van der Graaf WT (2014). First-line anthracycline-based chemotherapy for angiosarcoma and other soft tissue sarcoma subtypes: pooled analysis of eleven European Organisation for Research and Treatment of Cancer Soft Tissue and Bone Sarcoma Group trials. Eur J Cancer.

